# RGAAT: A Reference-based Genome Assembly and Annotation Tool for New Genomes and Upgrade of Known Genomes

**DOI:** 10.1016/j.gpb.2018.03.006

**Published:** 2018-12-21

**Authors:** Wanfei Liu, Shuangyang Wu, Qiang Lin, Shenghan Gao, Feng Ding, Xiaowei Zhang, Hasan Awad Aljohi, Jun Yu, Songnian Hu

**Affiliations:** 1 CAS Key Laboratory of Genome Sciences and Information, Beijing Institute of Genomics, Chinese Academy of Sciences, Beijing 100101, China; 2 Joint Center for Genomics Research (JCGR), King Abdulaziz City for Science and Technology and Chinese Academy of Sciences, Riyadh 11442, Saudi Arabia; 3 University of Chinese Academy of Sciences, Beijing 100049, China; 4 Grail Scientific Co. Ltd., Shenyang 110000, China; 5 Shenzhen Institute of Geriatrics, Shenzhen 518020, China

**Keywords:** Variant identification, Genome assembly, Genome annotation, Genome comparison

## Abstract

The rapid development of high-throughput sequencing technologies has led to a dramatic decrease in the money and time required for *de novo* genome sequencing or genome resequencing projects, with new genome sequences constantly released every week. Among such projects, the plethora of updated genome assemblies induces the requirement of version-dependent annotation files and other compatible public dataset for downstream analysis. To handle these tasks in an efficient manner, we developed the reference-based **genome assembly** and annotation tool (RGAAT), a flexible toolkit for resequencing-based consensus building and annotation update. RGAAT can detect sequence variants with comparable precision, specificity, and sensitivity to GATK and with higher precision and specificity than Freebayes and SAMtools on four DNA-seq datasets tested in this study. RGAAT can also identify sequence variants based on cross-cultivar or cross-version genomic alignments. Unlike GATK and SAMtools/BCFtools, RGAAT builds the consensus sequence by taking into account the true allele frequency. Finally, RGAAT generates a coordinate conversion file between the reference and query genomes using sequence variants and supports annotation file transfer. Compared to the rapid annotation transfer tool (RATT), RGAAT displays better performance characteristics for annotation transfer between different genome assemblies, strains, and species. In addition, RGAAT can be used for genome modification, **genome comparison**, and coordinate conversion. RGAAT is available at https://sourceforge.net/projects/rgaat/ and https://github.com/wushyer/RGAAT_v2 at no cost.

## Introduction

With the development of sequencing technologies, it is getting easier to obtain the genome of various species. Up to, genome sequences of 4963 eukaryotes, 125,679 prokaryotes, 12,952 viruses, 10,916 plasmids, and 10,965 organelles have been available in the NCBI genome database (https://www.ncbi.nlm.nih.gov/genome/browse#!/overview/; accessed on December 5, 2017) [1]. The sequence error rate is around 0.01% in the human genome [2]. However, the quality of genome sequences varied considerably due to a variety of factors such as different sequencing platforms used, even if improved by subsequent efforts, especially using next-generation sequencing platforms. In addition, some assemblies have obvious sequencing errors caused by the sequencing platform used, such as homopolymers from Roche/454 and base substitutions from Solexa [3]. Moreover, many more genome projects have released one reference assembly and several resequencing data for different cultivars or closely related species [4], [5]. The reference sequences are also constantly updated with newly emerging methods or strategies, such as 10X genomics long reads (https://www.10xgenomics.com/), single molecular sequencing (https://www.pacb.com/), and optical scan (https://bionanogenomics.com/). Thus, to maintain and utilize the different assemblies, genome upgrade, assembly, and annotation based on known assemblies are on common and great demands. Unfortunately, there are few easy-to-use integrated tools to achieve both genome assembly and annotation transfer based on known reference genomes. Despite some tools, such as SAMtools/BCFtools and GATK, containing the module to create consensus sequence, none of them considers the true allele frequency for each variant, which is important for reducing false positive rate [6], [7], [8], [9]. Another tool, rapid annotation transfer tool (RATT), can be used for annotation transfer, but the accuracy is relatively low for repeat regions [10], whereas iCORN can be used for correcting sequence errors, but not for upgrading annotations [11]. The web-based platforms—UCSC (https://genome.ucsc.edu/cgi-bin/hgLiftOver) and Galaxy (http://usegalaxy.org)—can convert coordinates among different genome assembly versions using the liftOver utility, but only for 106 genomes present in their databases [12], [13], [14], [15]. There is an increasing demand for genome comparison between sub-species and cultivars on the gene level. Therefore, it is imperative to achieve both reference-based genome assembly and annotation transfer for comparative genomic analysis. Unfortunately, there were few integral tools to perform both functions.

In this study, we reported the development of the reference-based genome assembly and annotation tool, RGAAT, to solve the problems encountered in the process of genome assembly and annotation. Although these problems are very common, we did not find comprehensive solutions despite searching two popular forums: Biostars (https://www.biostars.org/) and SEQanswers (http://seqanswers.com/). RGAAT is implemented in Perl and freely available to users at https://sourceforge.net/projects/rgaat/ and https://github.com/wushyer/RGAAT_v2. It accepts inputs of the genome sequence (FASTA format), annotation (GTF, GFF, GFF3, and BED format), mapping-based new assembly features, such as sequence alignment (SAM/BAM format), sequence variant (VCF format or tab-delimited five-column table containing chromosome, position, ID, reference allele, and alternative allele), and the new genome sequence (FASTA format). The search output displays sequence variants (for sequence alignment and genome comparison), updated genome sequence (for sequence alignment and sequence variant), corresponding coordinates between two genomes (known genome and upgrade/new genome), new genome annotation, and result of genome comparison. This tool can also be used to identify genome variants and to build genome consensus sequences.

## Method

**Figure 1:**
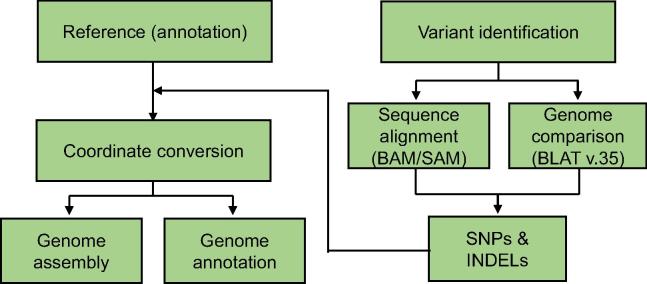
**Workflow of RGAAT** RGAAT includes three main modules: variant identification, coordinate conversion, and genome assembly/annotation. Variant identification is based on the sequence alignment or genome comparison result; annotation transfer is based on the processing coordinate conversion with variant calling result. The output of RGAAT includes new genome assembly and annotation.

RGAAT includes three main modules: variant identification, coordinate conversion, and genome assembly/annotation. The workflow of RGAAT is shown in Figure 1.

### Variant identification based on read alignment

**Figure 2:**
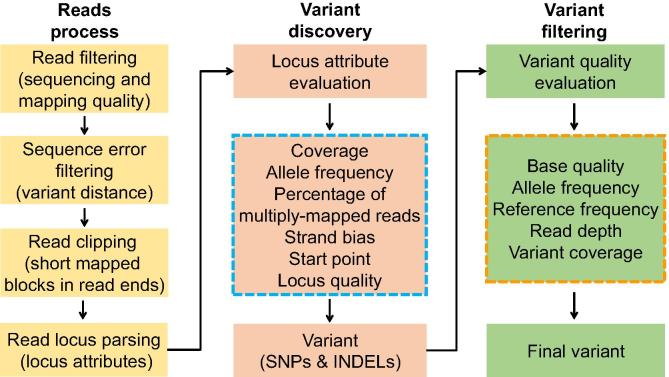
**Workflow of variant identification based on sequence alignment (SAM/BAM) in RGAAT** Variant identification based on sequence alignment includes 3 stages: read processing, variant discovery, and variant filtering. During the variant discovery stage, RGAAT applies a combination of criteria listed in the box with dashed borders in blue to increase the sensitivity of identification. During the variant filtering stage, RGAAT applies a combination of criteria listed in the box with dashed borders in orange to ensure the accuracy of identification. Finally, all candidate variant related attributes are recoded to reduce the false positive rate.

The principle of variant identification involves assessment of read quality, mapping quality, and sequence coverage. As several read mapping software have been developed to deal with read and mapping quality, we adopt the mapping results and handle the data at two stages: read processing and variant identification/filter (Figure 2).

**Supplementary Figure S1:**
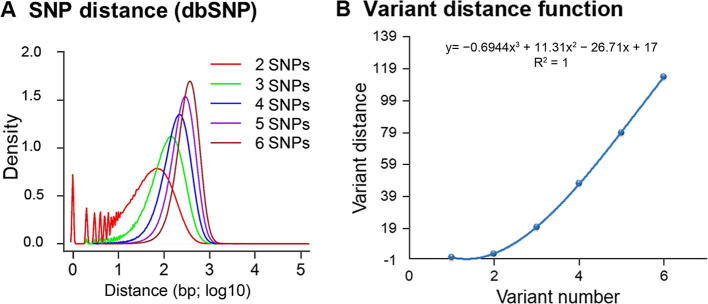
**The evaluation of sequence error and variant A.** Density plot of the distance between adjacent variants (bp) in dbSNP was used to set up the variant distance. Numbers before “SNPs” in the legend box indicate the numbers of consecutive SNP loci. **B.** Trinomial function (y = −0.6944x^3^ + 11.31x^2^ − 26.71x + 17; x and y indicate the variant number and variant distance, respectively) is used to filter reads based on read mismatch, insertion, and deletion to distinguish the true SNPs and sequencing errors.

The first part is read processing, *i.e.*, read filtering and locus parsing. Firstly, low-quality reads with average quality score <Q20 were abandoned. Then, we filtered the reads, including those with lower mapping quality, those from PCR or optical duplicate, and those with multiple mapping loci. To distinguish sequence error and variant, we evaluated the adjacent variant distance in dbSNP and built a variant distance trinomial function (*y* =  −0.6944*x*^3^ + 11.31*x*^2^ − 26.71*x* + 17, *x* and *y* indicate the variant number and variant distance, respectively) (Figure S1). Using this function, we filtered reads based on read mismatch, insertion, and deletion. Due to the relatively low accuracy for short mapped blocks at both ends of the read, we clipped the read ends with short match regions (length of cigar “M” <8 bp). After obtaining high-quality mapped reads, we parsed reads at each genome locus with quality check and masked reads in repeat regions that were handled by RepeatMasker (http://www.repeatmasker.org) and Tandem Repeats Finder [16]. Next, we recorded related attributes for each locus simultaneously, including (1) raw read coverage, (2) high-quality read coverage, (3) reference and alternative alleles, (4) base quality, (5) read start point number, (6) multi-mapped read number, and (7) mapped read number on each strand.

**Supplementary Figure S2:**
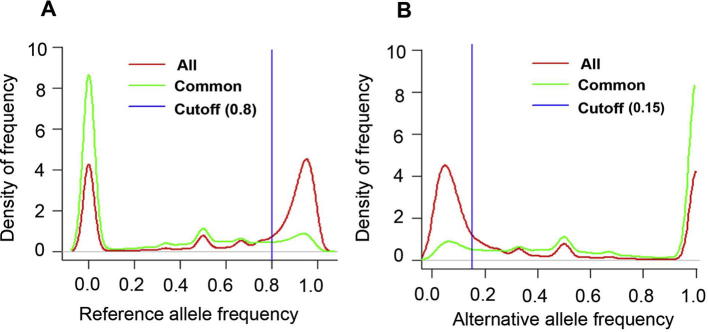
**The density of allele frequency** The density of reference allele frequency and alternative allele frequency is shown in **A** and **B**, respectively. “All” means all candidate variants, “Common” means common variants in dbSNP. To reduce the false positive rate of variant identification, cutoff values were set to 0.8 for reference allele frequency and 0.15 for alternative allele frequency. Variants with frequency >0.8 for reference allele and <0.15 for alternative allele were removed.

The second part is variant discovery and filtering. We firstly identified all candidate variants and recorded related attributes. To reduce the false positive rate, we removed the variants with low average base quality (<20), low uniquely mapped allele frequency (<15%), high reference allele frequency (≥80%), low read depth (<2), and low variant read number (<2) (Figure S2).

### Evaluation of identified variants based on four test datasets

To evaluate the efficiency and accuracy of RGAAT, we performed variant calling for four NGS datasets. These include Illumina 100 bp paired-end 30× exome data from the Genome Comparison And Analytic Testing (GCAT) toolkit, Illumina 101 bp paired-end 200× human exome data, as well as Illumina 101 bp paired-end data for plant chloroplast (∼5167×) and mitochondria (∼1788×) (Table 1), which were generated from other sequencing projects in our laboratory and deposited to GSA [17] under the accession Nos. PRJCA001139 (human exome data) and PRJCA000261 (chloroplast and mitochondria), respectively. After removing adaptor sequences and low-quality reads by Trimmomatic (v0.33) [18], filtered reads were mapped using Bowtie2 (v2.2.4) [19] with default setting. Variants were identified using GATK (duplicates marked by Picard Tools v1.119; INDEL intervals created by RealignerTargetCreator; reads realigned by IndelRealigner; and variants called by HaplotypeCaller of GATK v3.3-0-g37228af) [9], SAMtools (SAMtools v1.2 with configuration of -d 100000, -L 100000, and -m 3, and BCFtools v1.2 with default setting), and RGAAT. The variants of Freebayes_Q40 [20] were obtained from GCAT as well.

**Table 1:** **The four HiSeq datasets analyzed in this study**

**Feature**	**Human**	**Human**	**Chloroplast**	**Mitochondria**
Data type	100-bp pair-end exome	101-bp pair-end exome	101-bp pair-end genome	101-bp pair-end genome
Data source	GCAT	PRJCA001139	PRJCA000261	PRJCA000261
Coverage	30×	77×	5167×	1788×
Mapping software	Bowtie2(2.2.4)	BWA(0.7.10-r789)	Bowtie2(2.2.4)	Bowtie2(2.2.4)
No. of mapped reads	17,884,489	60,821,606	9,402,591	9,363,153
No. (percentage) of mapped clean reads	17,167,791 (95.99%)	59,862,730 (98.42%)	9,338,188 (99.32%)	9,198,701 (98.24%)
No. of raw variants	789,170	1,182,110	157,076	488,918
No. of variants after the first filtering	189,106	273,382	223	742
No. of final filtered variants	123,660	201,100	221	742

*Note*: dbSNP for human samples and the manually-curated variants for chloroplast and mitochondria sequences were used for evaluating the performance of variant calling in RGAAT. During read filtering step, unmapped reads, multi-mapped reads, reads generated from PCR duplicate, reads with low quality, high mismatch, chromosome difference, or large distance for paired-end were removed. At the first filtering step, variants with low read average base quality, low uniquely-mapped allele frequency, high reference frequency, low read depth, or low variant read number were removed. Variants of low read depth, low allele frequency, or low average read quality were filtered to obtain the final variants. GCAT, Genome Comparison and Analytic Testing platform.

### Variant identification based on genome comparison

**Figure 3:**
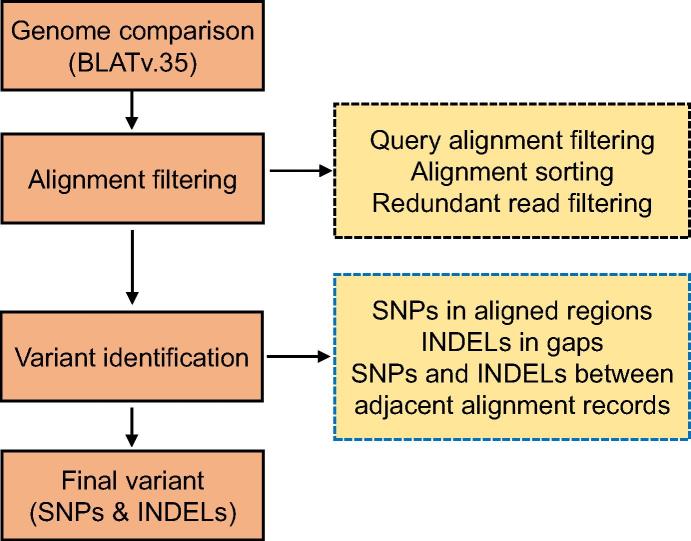
**Workflow of variant identification based on genome comparison in RGAAT** First, the genome comparison is performed by BLAT and the redundant reads are filtered out by the combination of processes indicated in the box with dashed borders in black. Second, variants including SNPs and INDELs are identified as indicated in the box with dashed borders in blue. Finally, variants were ready for downstream analysis.

RGAAT can be used to generate variants between two assemblies by sequence comparison (Figure 3). We used BLAT for genome comparison because of its ability to map sequences with long gap tolerance to eliminate the influence of repeat sequences, especially for different genome assemblies of the same species. First, we obtained the genome alignment using BLAT (v35) [21] with default setting. For genome comparison between different species, we used parameter “-minIdentity = 50” for BLAT. There were some redundant alignments and alignment errors in BLAT results due to the presence of repetitive and low-complexity regions. Based on the base number for match, mismatch, insertion, and deletion in query and target genome, we filtered the BLAT results step-by-step as follows. We first identified and kept the best alignment result for each query sequence; we then sorted query alignments based on the coordinate order in target sequences; and finally we removed bad alignments for overlapping records, that is, only the alignment with highest identity was kept whereas other alignments were removed. After that, we identified variants (SNPs and INDELs) and created genome coordinate conversion files (“TargetChrom, TargetStart, TargetEnd, QueryStart, QueryEnd, QueryChrom, QueryStrand”) based on the non-redundant genome alignment. Variants were identified at three levels, *i.e.*, SNPs in aligned regions, INDELs in gaps, as well as SNPs and INDELs located in the gaps of adjacent BLAT alignment records. The variants and coordinate conversion files are used in the downstream analysis.

### Consensus sequence building based on variants

One of the most common needs for re-sequencing projects and genome sequencing of closely-related cultivars, strains, or species is to reconstruct the new assembly based on read alignment files, such as the population-specific consensus genome sequences in humans [22] and other model species. Although GATK and SAMtools/BCFtools can build the consensus sequences based on variants, both tools have some disadvantages. First, GATK and SAMtools identify variants with suppressed read depth (500× coverage for GATK with the down-sampling setting for and maximally 250× coverage for SAMtools by default), which may affect allele frequency due to information loss with highly excessive coverage. Second, both tools create consensus sequences with non-reference alleles without considering the true allele frequency and read depth, which are very important parameters for genome upgrade. RGAAT improved the consensus sequence building in both aspects, that is, not setting read depth limit for variant identification and taking allele frequency into consideration. RGAAT can build consensus sequences easily by parsing the variant files in two steps, including (1) selecting the main allele among reference and alternative alleles and (2) adjusting genome location according to variants. For the first step, we selected the major allele based on the allele frequency. Software such as GATK and Freebayes can provide the allelic depths for the reference and alternative alleles by the allelic depth (AD) ID. RGAAT reports the exact allele read number and frequency of the reference and major alternative alleles during variant calling. Note that the “AF” ID in the VCF file is the max-likelihood estimate of the alternative allele frequency, which is not the true allele frequency. For the second step, we produced the genome coordinate conversion file for further annotation transfer.

### Annotation transfer based on variants or genome comparisons

**Figure 4:**
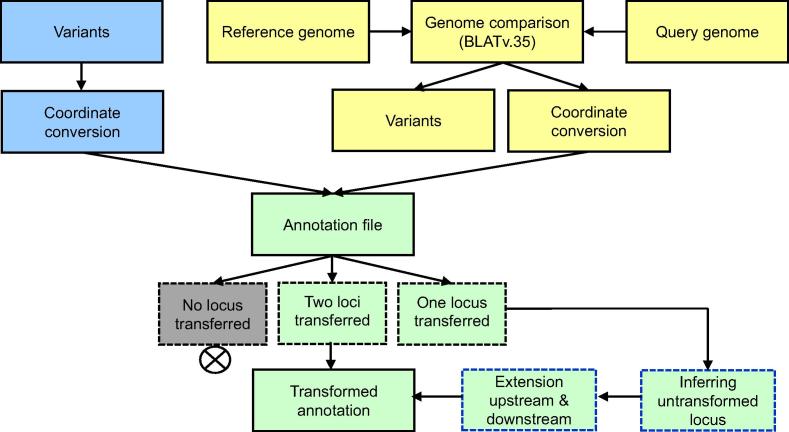
**Workflow of annotation transfer** The annotation transfer pipeline integrates two sets of information: the transfer progress based on variant identification (blue), and the transfer progress based on genome comparison (yellow). The integrated annotation file is classified into three categories (dashed borders in black): no locus transferred, two loci transferred, and one locus transferred. Annotation file in the first category is discarded, while file in the second one is transferred directly and file in the third one needs two extra steps (dashed borders in blue) to infer untransformed locus by extension to both upstream and downstream regions.

In addition to new genome creation, annotation transfer is an important step for assembly upgrade and further genome comparison at the gene level. Application of next-generation sequencing (NGS) technologies has greatly reduced the sequencing cost and promoted the productivity of genome sequencing projects dramatically. However, genome annotation is both arduous and computing-intensive. Several automatic annotation tools, such as Ensembl [23], NCBI [1], PASA [24], and MAKER [25] have been developed. However, these complicated tools require expertise to use and are more suitable for *ab initio* genome annotation. RATT is a tool for annotation transfer between similar genomes and can be run easily and quickly [10]. However, RATT uses MUMmer [26] as aligner, resulting in the loss of global sequence consistency during alignment for closely-related genomes, especially for repeat regions. For rapid upgrade genome annotation between different genome assemblies, RGAAT can build genome coordinate conversion files based on variants or genome comparison. There are two options for genome annotation transfer: one is to replace the reference genome with variants (creating a new consensus sequence) and change the coordinate for corresponding annotation files; and the other is to transfer reference annotations to the target genome based on genome comparison (Figure 4). The former is suitable for genome upgrade, while the latter performs better for closely-related genomes without re-sequencing data.

It is important to define the exact syntenic regions between the two assemblies in annotation transfer. With the genome coordinate conversion file, the coordinates of annotation features in the reference genome are transformed. For each annotation feature, the outcome of annotation transfer can be classified into three groups according to the status of start/end locus: two loci successfully transferred, one locus successfully transferred, and no locus transferred. In the first case, two loci can be easily replaced with new genome coordinates. In the second case, the non-transformed locus can be inferred from the successfully-transferred locus, by considering the distance of two loci in the reference genome and the strand information in the query genome. To reduce the influence of syntenic loss in the low identity region, RGAAT tries to find possible start or stop codons by extending to upstream or downstream sequences in order to infer the non-transformed site. For the first exon, RGAAT tries to find the possible start codon by extending upstream, while for the last exon, RGAAT tries to find the possible stop codon by extending downstream. Due to sequence variations between the two genomes, annotation features may be interfered by SNPs and INDELs, especially for coding sequences (CDS). Meanwhile, to achieve the maximum annotation transfer in syntenic regions, we keep all candidate annotations in the output files and mark the location of problematic annotations (annotations partially transferred due to the interruption by the presence of stop codons) using an interrogation sign “?”. Users can check the annotation with “?” markers to recover partially transferred annotations interrupted by stop codons. In addition, we prefer using the feature table file (*.tbl) for annotation transfer to be compatible with the NCBI record system. However, it should be pointed out that the success in direct transfer of genes highly relies on the similarity between the two genomes. For the annotation of problematic features, we refer to the information from the successfully-transferred locus, including distance between the two loci of the feature in the reference genome and their strandness in the query genome to ensure the completeness of ORFs. If the similarity of genome is too low, the results of annotation transfer would become unpredictable. In this case, we highly recommend a fully *ab initio gene* prediction for very distinct genomes.

### Evaluation of annotation transfer between genomes in five datasets

We evaluate the efficiency of annotation transfer using RGAAT based on five datasets. These include two chloroplast genome assemblies generated in our lab (GenBank accession: KX028884) using different sequencing platforms, 454 and Solexa, which includes corrected 212 regions in total consisting of 119 base errors, 6 deletions, and 87 insertions as reference to assess annotation transfer between different genome assemblies. To evaluate the annotation transfer between strains, the bacterium *Mycobacterium tuberculosis* (strain H37Rv; GenBank accession: AL123456 and strain F11; GenBank accession: CP000717) and chromosome IV of yeast *Saccharomyces cerevisiae* (strain S288C; GenBank accession: NC_001136 and strain ySR127 GenBank accession: CP011550) were used. In addition, the chromosome 14 of the parasites *Plasmodium chabaudi* and *P. berghei* (downloaded from http://ratt.sourceforge.net) and the chromosome IV of yeast *S. cerevisiae* (strain S288C; GenBank accession: NC_001136) and *S. arboricola* (strain H-6; GenBank accession: CM001566) were used to assess annotation transfer between species.

### SVG figure creation for genome comparison

To reveal the variations between two genomes, an SVG figure is created for each chromosome based on the genome coordinate conversion file and genome annotation file. The figure shows SNPs, INDELs, and identical regions with different colors for the compared genomes with gene blocks. SVG file can be displayed easily in browser, using functions such as drag, zoom in, and zoom out.

## Results

Here, we demonstrate the performance of RGAAT, mainly in two parts: variant identification and annotation transfer.

### Variant identification

#### For dataset with low sequence depth

The 30× 100 bp paired-end exome dataset was downloaded from GCAT website and aligned using Bowtie2 with default parameters (Table 1). Before variant identification, we filtered 4.01% aligned reads including those with low sequence quality (3.16%), low mapping quality (0.06%), and high percentage of mismatches (0.79%). We also filtered the short aligned regions on both end of the reads. From the retained mapped reads, we identified 789,170 raw variants. After examining the base quality, variant frequency, reference frequency, multi-mapped reads, read start count, read depth, and the discrepancy between the numbers of reference and variant reads, only 189,106 (23.96%) variants passed the initial filter criteria. Then, we filtered the variants according to the percentage and quality of variant reads. In total, 123,660 final variants were identified. For comparison, GATK and SAMtools were also used to identify variants simultaneously. We uploaded the variants identified by RGAAT, GATK, and SAMtools to GCAT and compared them together with Freebayes. According to the comparison reports, the precision rate of variant identification using RGAAT is higher than those using Freebayes and SAMtools, and was similar to that using GATK. As for sensitivity, the performance of RGAAT is comparable with that of GATK, but higher than that of Freebayes and lower than that of SAMtools (Table 2). Upon validation with dbSNPs, we observed that RGAAT identified higher number of common variants than GATK, and lower number of novel variants than SAMtools and Freebayes. These observations indicate that RGAAT achieves a good balance between true positives and false positives, which is consistent with the precision rate, specificity, and sensitivity exhibited by RGAAT. In addition, RGAAT shows a higher transition/transversion ratio (Ti/Tv; the number of transitions to the number of transversions for SNP variants; 2.156) than Freebayes (1.826) and SAMtools (1.483), which is comparable with GATK (2.356).

**Table 2:** **Comparison of performance in variant identification using different tools**

**Dataset**	**Software**	**TP**	**FP**	**TN**	**FN**	**Precision**	**Sensitivity**	**Specificity**
Human (GCAT)^a^	RGAAT	20,032	1432	46,467,105	3471	93.33%	85.23%	100.00%
	GATK	19,861	1121	46,467,416	3321	94.66%	85.67%	100.00%
	SAMtools	20,537	3227	46,465,310	2248	86.42%	90.13%	99.99%
	Freebayes_Q40	18,925	1854	46,466,683	4376	91.08%	81.22%	100.00%

Human (GCAT)^b^	RGAAT	14,487	468	59,286	2045	96.87%	87.63%	99.22%
	GATK	14,180	732	59,311	2063	95.09%	87.30%	98.78%
	SAMtools	14,862	802	59,230	1392	94.88%	91.44%	98.66%
	Freebayes_Q40	13,688	555	59,321	2722	96.10%	83.41%	99.07%

Human (GSA)^c^	RGAAT	38,527	785	48,223,655	2,926,351	98.00%	1.30%	100.00%
	GATK	42,773	1553	48,222,887	2,922,105	96.50%	1.44%	100.00%
	SAMtools	41,963	1882	48,222,558	2,922,915	95.71%	1.42%	100.00%

Chloroplast^d^	RGAAT	198	23	154,300	14	89.59%	93.40%	99.99%
	GATK	163	123	154,200	49	56.99%	76.89%	99.92%
	SAMtools	181	83	154,240	31	68.56%	85.38%	99.95%

Mitochondria^e^	RGAAT	624	118	677,331	60	84.10%	91.23%	99.98%
	GATK	560	238	677,211	124	70.18%	81.87%	99.96%
	SAMtools	581	220	677,229	103	72.53%	84.94%	99.97%

*Note*: The variants of NIST Genome in a Bottle (GIAB, a) and the variants of Illumina OMNI SNP Array (b) were obtained from GCAT; the dbSNP variants for human exome (c) were deposited in GSA (accession No. PRJCA00113); the manually curated variants for chloroplast (d) and mitochondria (e) were deposited in GSA (accession No. PRJCA00261). TP, true positive; FP, false positive; TN, true negative; FN, false negative.

#### For dataset with high sequence depth

To assess the performance of identification at higher read depths, we applied RGAAT, GATK, and SAMtools to identify variants in one medium-depth data (200× human exome dataset) and two high-depth data (5167× of chloroplast and 1788× of mitochondria datasets) (Table 2). With 200× human exome data, RGAAT showed the highest precision rate and specificity, but lowest sensitivity. The Ti/Tv ratios were 2.63, 2.31, and 2.28 for SAMtools, RGAAT, and GATK, respectively. For the two high-depth data, RGAAT displayed better performance, *i.e.*, higher precision rate, sensitivity, and specificity than SAMtools and GATK (Table 2).

#### Variant identification by genome comparison

**Supplementary Figure S3:**
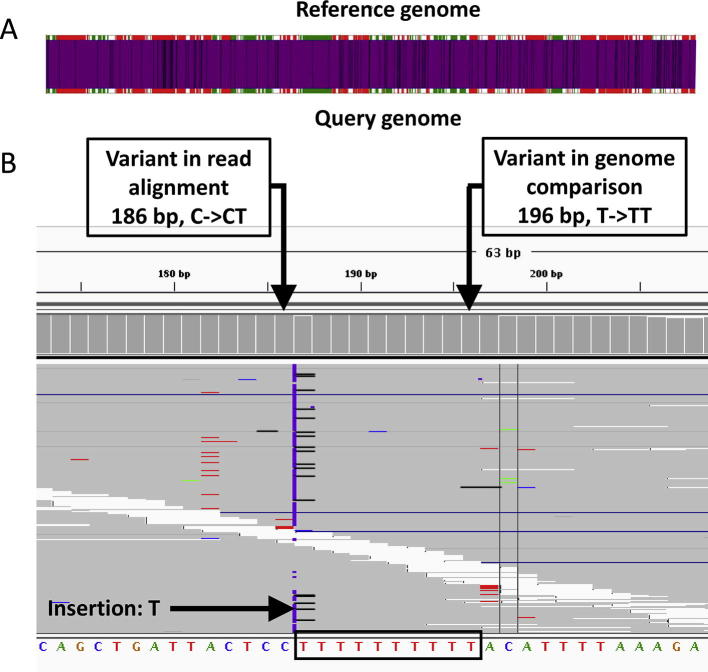
**Variant identification by genome comparison A.** The pairwise synteny of query and reference genomes. Genes and repeats are colored in red and green, respectively, with gene linkages shown in purple. Variants were identified based on the comparison. **B.** The variant position difference between read alignment and genome comparison by BLAT. The variant loci were identified by BLAT (right) and the variant position were identified from read alignment (left).

We performed sequence alignment to identify variants between two versions of chloroplast genome sequence generated in our lab using two different platforms by BLAT and compared them with the true variants. All 212 true variants (119 SNPs, 87 insertions, and 6 deletions) were identified by genome comparison, including 191 one-to-one, 8 two-to-one, and 1 five-to-one variant matches (Figure S3A). Note that the variants identified by BLAT is located at the end of aligned region, while the variant identified from read alignment is located in the start of aligned region (see Figure S3B for example).

### Annotation transfer

#### Annotation transfer between different genome assemblies

We obtained two genome assembly versions for the chloroplast sample. Using two annotation transfer methods in RGAAT, *i.e.*, variant-based and genome comparison-based, all annotation features were successfully transformed, including 93 CDSs, 54 exons, 141 genes, 8 rRNAs, and 40 tRNAs. In comparison, RATT, another annotation transfer tool, lost 8 genes, 14 CDSs, 1 exon, and 8 tRNAs during transfer (Table 3). In particular, the transferred annotation in RATT contained one partial CDS and two frameshift CDSs (Table S1).

**Table 3:** **Comparison of performance in annotation transfer using RGAAT and RATT**

**Scenario**	**Samples**	**No. of reference features**	**No. of target features**	**Tool**	**No. of transferred features**	**No. of problematic features**	**Comparison with known annotation**				
							**TP**	**FP**	**FN**	**Precision**	**Sensitivity**
Between different genome assemblies	Chloroplast	336	336	RGAAT	336	0	336	0	0	100.00%	100.00%
				RATT	305	3	303	2	33	99.34%	90.18%

Between different strains	*M. tuberculosis*	8540	7996	RGAAT	8417	29	7796	318	198	96.08%	97.50%
				RATT	8223	41	7648	314	348	96.06%	95.65%
	*S. cerevisiae*	2430	-	RGAAT	2430	5	-	-	-	-	-
				RATT	2430	22	-	-	-	-	-

Between different species	*P. chabaudi*/*P. berghei*	686	661	RGAAT	652	186	636	16	25	97.55%	96.22%
				RATT	647	174	633	14	28	97.83%	95.76%
	*S. cerevisiae/S. arboricola*	2430	1106	RGAAT	1401	105	670	671	436	49.96%	60.58%
				RATT	993	13	661	321	445	67.31%	59.76%

*Note*: The number of reference features is the number of annotations from the source genome; the number of target features is the number of reference annotations already available on the targeted genome; the number of transferred features is the number of annotations transferred by software based on the annotation of the source genome and the comparison of the two genome sequences; and the number of problematic features is the number of annotations partially transferred due to the interruption by the presence of stop codons. Parameters including TP, FP, FN, precision and sensitivity are calculated based” on the number of transferred features and the number of target features. “-” indicates that there is no this kind of feature in query genome and the number of FP is overestimated due to the inclusion of pseudo genes. TP, true positive; FP, false positive; FN, false negative.

#### Annotation transfer between different strains

First, we tested the annotation transfer from the bacterium *Mycobacterium tuberculosis* strain H37Rv to the strain F11 genome because these two closely related genomes are relative well assembled and annotated. Both RATT and RGAAT completed the transfer within several minutes. Of 8540 annotation features in strain H37Rv, 8417 (98.56%) and 8223 (96.29%) were transferred to F11 by RGAAT and RATT, respectively (Table 3 and Table S2). We inspected all CDSs of strain F11 and found that only 29 (0.73%) in RGAAT and 41 (1.05%) in RATT were not transferred correctly. Among them, in-frame stop codons were found in the translation of 17 and 15 CDSs in RGAAT and RATT, respectively, indicating that these CDSs could be pseudogenes. Comparing with known annotation of F11, RGAAT shows similar precision rate (96.08%) with RATT but higher sensitivity (97.50%). Moreover, 140 (4 problematic) and 137 (8 problematic) novel CDSs were identified by RGAAT and RATT, respectively. We then used the chromosome IV of yeast strain S288C to annotate the strain ySR127 that was submitted to NCBI without annotation. All 2430 annotation features in strain S288C were successfully transferred by both RGAAT and RATT (Table 3 and Table S3). Among them, the translation of 5 CDSs in RGAAT and 22 CDSs in RATT terminates earlier, most of which were transferred incorrectly. We thus compared the annotation results between RGAAT and RATT and found that 4 mobile elements and 20 CDSs were inconsistently annotated. After comparing with repetitive elements, we found that 4 mobile elements were mis-transferred in RATT. Among the 20 discrepant CDSs, 18 was incorrectly transferred in RATT, which led to frame shift, and the remaining 2 terminated earlier due to the stop codons present in RGAAT.

#### Annotation transfer between different species

We also evaluated the performance of RGAAT for annotation transfer between two closely related eukaryote species in two datasets since accuracy and sensitivity of annotation transfer directly affect downstream gene function analysis. In the previous report on RATT [10], the *P. chabaudi* was used to annotate *P. berghei* chromosome 14. Our test showed that, using this dataset, 652 and 647 out of 686 reference CDSs were transferred from chromosome 14 of *P. chabaudi* to that of *P. berghei* by RGAAT and RATT, respectively, of which 186 and 174 CDSs terminated earlier by stop codon in RGAAT and RATT, respectively (Table 3). When we checked the translation of *P. chabaudi* CDSs [10], we found that 470 CDSs were interrupted by in-frame stop codons. The bad quality of reference annotation made it difficult to perform further comparisons.

Meanwhile, genome information of *S. cerevisiae* chromosome IV was used to annotate *S. arboricola* chromosome IV. 1401 and 993 of 2430 reference annotation features were transferred by RGAAT and RATT, respectively, with 105 and 13 CDSs containing multiple in-frame stop codons, respectively (Table 3 and Table S4). We analyzed the 105 CDSs in RGAAT and found 10 of them seemingly pseudogenes. Comparing with RATT, RGAAT has higher sensitivity and lower precision rate. The main reason for low precision rate in RGAAT is the higher number of transferred features in RGAAT compared to RATT (1401 *vs*. 993). Additionally, the original annotated features may include pseudogenes, which was removed in the RGAAT, leading to the underestimated precision rate. If the problematic features were removed, the precision rate would be higher. False negative annotation features could be further recovered from problematic features by manual inspection.

## Other applications

In addition to the functions described above, RGAAT can be used for other applications. For instance, RGAAT can read the tab delimited text file with five columns (chromosome, position, ID, reference allele, and alternative allele) and build new genome assembly based on alternative alleles, which means that user can edit genome sequences (insertion and deletion) easily by just providing the edit position and sequence. In addition, based on sequence comparison, RGAAT can identify variants between two genomes and evaluate the influence of these variants using other downstream analysis tools such as ANNOVAR [27]. RGAAT can also provide the SVG graphical results for assembly comparison based on coordinate conversion file and genome annotation files.

## Conclusions

RGAAT is an efficient tool for assembly upgrade and annotation transfer to new assembly based on known reference genomes. The variant identification for human exome sequencing can be achieved in less than one day using one CPU and approximately 16 Gb memory on a Linux system. RGAAT is compatible with many variant input types: (1) tab delimited text file provided by users with five columns (chromosome, position, ID, reference, and variant); (2) variant call format file created by other software, such as GATK, SAMtools, and Freebayes; (3) sequence alignment file provided in SAM or BAM format; and (4) new genome sequences provided in FASTA format. Compared with GATK, SAMtools, and Freebayes, RGAAT and GATK have similar precision rate (TP/(TP + FP)) and specificity (TN/(TN + FP)), but exhibit slightly lower sensitivity (TP/(TP + FP)) for the NIST Genome in a Bottle dataset (GCAT) (https://www.nist.gov/programs-projects/genome-bottle), whereas RGAAT shows the highest precision rate and specificity on Illumina OMNI SNP Array, indicating that RGAAT achieves a good balance between true and false positives. In addition, RGAAT has a higher Ti/Tv ratio than Freebayes and SAMtools, which is comparable with GATK. A higher Ti/Tv ratio generally suggests high accuracy in our variant calling test for human exome [9]. To build consensus sequences, we parse all reads for variant identification and consider the true allele frequency for variant selection. RGAAT has better performance for different genome assemblies and strains (>96% precision rate and sensitivity), although the annotation transfer is influenced by sequence similarity between two species. Compared to RATT, RGAAT has higher transfer percentage, higher sensitivity and lower problematic annotation percentage. RGAAT can also support some popular annotation formats such as GTF, GFF, GFF3, and BED (Table S5). Although we provide some frequently used modules for genome assembly and annotation, there remains much work to be further optimized. RGAAT is implemented in PERL and tested in Linux environments. The detailed description can be found in the README file of RGAAT software package.

In summary, RGAAT provides several functional modules for handling frequently-used genome analysis, such as genome variant identification, genome consensus sequence building, genome modification, genome comparison, and annotation transfer. RGAAT will benefit the comparative genomic analysis between closely-related species and sub-species at the gene level, such as pan-genome analysis and population genetics.

## Authors’ contributions

HAA, JY, and SH conceived the study; WL, SW, and QL wrote the codes. WL and QL wrote the manuscript; SG, FD, and XZ revised the manuscript. All author read and approved the manuscript.

## Competing interests

The authors have declared no competing interests.
