# Supplementary material for: RGAAT: A Reference-based Genome Assembly and Annotation Tool for New Genomes and Upgrade of Known Genomes
Source: Genomics Proteomics Bioinformatics. 2018 Dec 21;16(5):373–81. doi: 10.1016/j.gpb.2018.03.006 (PMC6364042; doi:10.1016/j.gpb.2018.03.006)
Supplement: Supplementary Table S1 [file mmc1.docx]

**Table S1 Annotation transfer between two chloroplast assemblies using RGAAT and RATT**

| **Feature** | **No. of reference features** | **Tool** | **No. of transferred features** | **No. of target features** | **Comparison with known annotation** | | | | |
| --- | --- | --- | --- | --- | --- | --- | --- | --- | --- |
|  |  |  |  |  | **TP** | **FP** | **FN** | **Precision** | **Sensitivity** |
| Gene | 141 | RGAAT | 141 | 141 | 141 | 0 | 0 | 100.00% | 100.00% |
|  |  | RATT | 133 |  | 132 | 1 | 9 | 99.25% | 93.62% |
| CDS | 93 | RGAAT | 93 | 93 | 93 | 0 | 0 | 100.00% | 100.00% |
|  |  | RATT | 79 |  | 78 | 1 | 15 | 98.73% | 83.87% |
| Exon | 54 | RGAAT | 54 | 54 | 54 | 0 | 0 | 100.00% | 100.00% |
|  |  | RATT | 53 |  | 53 | 0 | 1 | 100.00% | 98.15% |
| rRNA | 8 | RGAAT | 8 | 8 | 8 | 0 | 0 | 100.00% | 100.00% |
|  |  | RATT | 8 |  | 8 | 0 | 0 | 100.00% | 100.00% |
| tRNA | 40 | RGAAT | 40 | 40 | 40 | 0 | 0 | 100.00% | 100.00% |
|  |  | RATT | 32 |  | 32 | 0 | 8 | 100.00% | 80.00% |

*Note*: The number of reference features is the number of annotations from the source genome; the number of target features is the number of reference annotations already available on the targeted genome, and the number of transferred features is the number of annotations transferred by software based on the annotation of the source genome and the comparison of the two genome sequences. Parameters including TP, FP, FN, precision and sensitivity are calculated based on the number of transferred features and the number of target features. The number of FP is overestimated due to the inclusion of pseudo genes. TP, true positive; FP, false positive; FN, false negative.
