# Supplementary material for: RGAAT: A Reference-based Genome Assembly and Annotation Tool for New Genomes and Upgrade of Known Genomes
Source: Genomics Proteomics Bioinformatics. 2018 Dec 21;16(5):373–81. doi: 10.1016/j.gpb.2018.03.006 (PMC6364042; doi:10.1016/j.gpb.2018.03.006)
Supplement: Supplementary Table S3 [file mmc3.docx]

**Table S3 Annotation transfer between two *Saccharomyces cerevisiae* strains using RGAAT and RATT**

| **Feature** | **No. of reference features** | **Tool** | **No. of transferred features** | **Comparison of RGAAT and RATT** | | |
| --- | --- | --- | --- | --- | --- | --- |
|  |  |  |  | **Common** | **Unique** | **Problematic** |
| Gene | 788 | RGAAT | 788 | 788 | 0 | 0 |
|  |  | RATT | 788 |  | 0 | 0 |
| CDS | 756 | RGAAT | 756 | 736 | 20 | 5 |
|  |  | RATT | 756 |  | 20 | 22 |
| mRNA | 755 | RGAAT | 755 | 740 | 15 | 5 |
|  |  | RATT | 755 |  | 15 | 22 |
| ncRNA | 4 | RGAAT | 4 | 4 | - | - |
|  |  | RATT | 4 |  | - | - |
| tRNA | 28 | RGAAT | 28 | 28 | 0 | - |
|  |  | RATT | 28 |  | 0 | - |
| Rep origin | 41 | RGAAT | 41 | 41 | 0 | - |
|  |  | RATT | 41 |  | 0 | - |
| Mobile element | 8 | RGAAT | 8 | 4 | 4 | 0 |
|  |  | RATT | 8 |  | 4 | 4 |
| LTR | 36 | RGAAT | 36 | 36 | 0 | - |
|  |  | RATT | 36 |  | 0 | - |
| STS | 6 | RGAAT | 6 | 6 | 0 | - |
|  |  | RATT | 6 |  | 0 | - |
| Centromere | 4 | RGAAT | 4 | 4 | 0 | - |
|  |  | RATT | 4 |  | 0 | - |
| Misc feature | 2 | RGAAT | 2 | 2 | 0 | - |
|  |  | RATT | 2 |  | 0 | - |
| Telomere | 2 | RGAAT | 2 | 2 | 0 | - |
|  |  | RATT | 2 |  | 0 | - |

*Note*: The number of reference features is the number of annotations from the source genome; the number of transferred features is the number of annotations transferred by software based on the annotation of the source genome and the comparison of the two genome sequences; and the number of problematic features is the number of annotations partially transferred due to the interruption by the presence of stop codons. “-“ indicates that there is no this kind of feature in query genome.
