# Supplementary material for: RGAAT: A Reference-based Genome Assembly and Annotation Tool for New Genomes and Upgrade of Known Genomes
Source: Genomics Proteomics Bioinformatics. 2018 Dec 21;16(5):373–81. doi: 10.1016/j.gpb.2018.03.006 (PMC6364042; doi:10.1016/j.gpb.2018.03.006)
Supplement: Supplementary Table S5 [file mmc5.docx]

**Table S5 Annotation transfer in four model species using RGAAT**

| **Species** | **Chromosome** | **Annotation format** | **Running time (s)** | **CPU number** |
| --- | --- | --- | --- | --- |
| *Caenorhabditis elegans /* *Caenorhabditis briggsae* | III | GFF3 | 1752 | 1 |
| *Drosophila melanogaster / Drosophila simulans* | 2R | GFF3 | 7212 | 1 |
| *Mus musculus /*  *Mus musculus domesticus* | 19 | GFF3 | 3559 | 32 |
| *Homo sapiens /*  *Pan troglodytes* | Y | GFF3 | 1150 | 32 |

*Note*: All genome sequences and annotation were downloaded from Ensembl. The annotation for chromosome III from *C. elegans* was transferred to *C. briggsae*; the annotation for chromosome 2R from *D. melanogaster* was transferred to *D. simulans*; the annotation for chromosome 19 from *M. musculus* was transferred to *M. musculus domesticus*; the annotation for chromosome Y from *H. sapiens* was transferred *to Pan troglodytes*.
